# Review of Neural Network Modeling of Shape Memory Alloys

**DOI:** 10.3390/s22155610

**Published:** 2022-07-27

**Authors:** Rodayna Hmede, Frédéric Chapelle, Yuri Lapusta

**Affiliations:** CNRS, Clermont Auvergne INP, Institut Pascal, Université Clermont Auvergne, F-63000 Clermont-Ferrand, France; frederic.chapelle@sigma-clermont.fr (F.C.); yuri.lapusta@sigma-clermont.fr (Y.L.)

**Keywords:** shape memory alloy, artificial neural network, SMA actuators, SMA sensors, SMA shapes, SMA properties

## Abstract

Shape memory materials are smart materials that stand out because of several remarkable properties, including their shape memory effect. Shape memory alloys (SMAs) are largely used members of this family and have been innovatively employed in various fields, such as sensors, actuators, robotics, aerospace, civil engineering, and medicine. Many conventional, unconventional, experimental, and numerical methods have been used to study the properties of SMAs, their models, and their different applications. These materials exhibit nonlinear behavior. This fact complicates the use of traditional methods, such as the finite element method, and increases the computing time necessary to adequately model their different possible shapes and usages. Therefore, a promising solution is to develop new methodological approaches based on artificial intelligence (AI) that aims at efficient computation time and accurate results. AI has recently demonstrated some success in efficiently modeling SMA features with machine- and deep-learning methods. Notably, artificial neural networks (ANNs), a subsection of deep learning, have been applied to characterize SMAs. The present review highlights the importance of AI in SMA modeling and introduces the deep connection between ANNs and SMAs in the medical, robotic, engineering, and automation fields. After summarizing the general characteristics of ANNs and SMAs, we analyze various ANN types used for modeling the properties of SMAs according to their shapes, e.g., a wire as an actuator, a wire with a spring bias, wire systems, magnetic and porous materials, bars and rings, and reinforced concrete beams. The description focuses on the techniques used for NN architectures and learning.

## 1. Introduction

Since the 1980s, a new and dynamically developing field has been emerging known as intelligent materials or smart materials (SMs). These materials play an essential role in various technological fields [1,2], such as aerospace and automobile [3], robotics [4], medical [5], engineering [6], actuators [7], sensors [8,9], and rotary manipulators [10]. Shape memory alloys (SMAs) are one of the most highlighted smart materials [11]. There are many types of SMAs, such as Fe–Mn–Si, Cu–Zn–Al, and Cu–Al–N, and each type has specific applications, but Nitinol Ni-Ti is one of the most used and studied due to its stability [12].

SMA is a raw material used in various fields. It can memorize its initial form upon cyclic mechanical transformations. These transformations depend on temperature and stress factors [13]. The two main properties: pseudoelasticity and shape memory effect, are owed to the usage of SMA as actuators, sensors, and controllers. Moreover, SMAs, such as the Nickel Inconel 718 alloy, have high corrosion and oxidation resistance, increasing the potential of SMAs in various fields such as gas turbines and jet engine applications [3,14]. To predict the properties of SMAs, their compounds, and their crystal structure, many applications [15] have been used depending on computational, experimental [16], and finite element methods (FEMs).

In addition, machining processes have been employed to analyze SMA behavior via dedicated conventional and nonconventional methods [17,18,19,20]. For example, in [14], they worked on optimizing the process’s factors of Laser Beam Machining (LBM). LBM is a machining process based on thermal energy, meaning that the machine removes the material by melting, vaporization, or chemical degradation. They proposed Taguchi–Grey Relation Analysis (TGRA) to provide the best quality cutting surface of the nickel Inconel 718 alloy. For that, they performed LBM’s measurements using a 2 KW power, 0.7 mm as a nozzle distance, 2 mm as a focal length, and 3 bar as gas pressure. Moreover, for the machining of nickel–titanium using wire-electrical-discharge-machining (WEDM) [3], the authors studied the optimized parameters using a 3D microscopic analyzer for having a defect-free surface and low globules. As a result, the parameter conditions are pulse-on time 65 µs, pulse-off time 32 µs, and discharge current 6 A. In [20], they used the heat-transfer search (HTS) algorithm that was effective in predicting and optimizing the input values of WEDM of the nickel–titanium. They proved the relationship between the input (pulse-on time, pulse-off time, and current) and output (material removal rate, surface roughness, and micro-hardness) variables. Furthermore, they proved the significance of the micro-hardness for the necessity of the shape memory effect (SME) possession after the machining to eliminate the risk of destroying the SMA. By that, we can highlight the significance of the post-processing of SMAs in any application. Post-processing aims to prevent the destruction of an SMA and to initialize the crystalline structure with the solid phase: austenite or martensite.

However, the nonlinear behavior of SMAs and high computational cost are obstacles to obtaining real-time simulations using these methods.

Artificial intelligence (AI) can be used to address the difficulties of modeling SMA behavior. AI has been known since 1955 due to a proposal authored by McCarthy, Minsky, Rochester, and Shannon [21]. This field has faced many transformations because of changes in the definition of intelligence over the past decades. Today, AI is a basis for modeling in robotics and machine fields and has achieved great success in materials science. Many types of research using AI have focused on studying materials by modeling their behavior and especially the commercially available materials, scanning their structure, simulating their deformation in real-time, and discovering new materials [22,23,24,25,26,27].

Machine learning is an important subset of AI [28] suitable for high-dimensional data problems with linear and nonlinear properties and has succeeded in many applications concerning smart materials [29,30]. Materials discovery is one of the widespread applications where artificial neural networks (ANNs), support vector machines (SVMs), and Bayesian methods can be used for designing new materials such as guanidinium ionic liquids [31] or crystals [32]. Material property prediction can be achieved by reinforcement learning (RL), ANN, general regression neural network (GRNN), and support vector regression (SVR) methods. This approach can be applied to applications such as constant lattice prediction [33]. Real-time simulation in the medical field can be performed by ANNs to model the human liver’s biomechanical behavior during the breathing process [34]. In [23], a deep learning method was utilized to study the behavior of hyperelastic materials in medical applications. AI was applied to different properties of smart materials, such as composite sandwiches (CSs). The bending strength (flexural strength) was predicted in [35]. The authors trained ANNs using the MATLAB platform and compared it to the experimental studies. These investigators tested the Levenberg–Marquardt (LM) algorithm for training and compared its results to those of the backpropagation algorithm. The validation of an ANN can depend on the values of performance, regression correlation (R), and mean square error (MSE). These authors highlighted the effect of the density of the CS. The CS can be differentiated according to the percentage of water absorbed as a function of time and temperature (moisture absorption). Hence, using NNs, they confirmed that the bending strength and the core shear strength should be reduced by 53% and 42.78%, respectively, for bending between 30° and 50°.

We are focusing on the SMAs owed to their potential, especially in the mechanic, robotic and medical fields. The motivation of this work is to fill the lack of citation of the potential of AI and especially the NN in modeling the behavior of SMAs. This is a promising technique used recently in the domain of smart materials. Therefore, in this review paper, we present these studies proving the ability of NNs to model the characteristics and properties of SMAs. We classify them according to the shape of the SMA, the type of application, the type of NN, and the training method. The aim is to give guidelines for the settings of NNs when they are used in SMAs’ applications. The shape of the SMA systems specifies their behavior and determines the type of the application’s problem. Consequently, NN’s type depends on the applied problem. Therefore, the current paper is organized to show the dependency between the type of NNs and the shape of SMAs. We classify them according to the shape of the SMA, the type of application, the type of the NN, and the training method. The next sections give an overview of the characteristics of SMAs (Section 2), describe the characteristics and types of the used NNs (Section 3), specify the application of NNs to model SMAs according to their shapes (Section 4), and discuss the specifications of this application to design an adapted NN model (Section 5). We conclude by giving the relevance of ANNs in modeling the behavior of SMAs (Section 6).

## 2. Description of the SMAs

Shape memory alloys are a well-known class in smart materials. These materials can hold large stresses without being permanently strained. The most known and interesting type of SMA is Nitinol (Ni-Ti) [36], which is a widespread choice for engineering processes in the medical [5,37], robotics [38,39], civil engineering [40,41], aerospace [42,43], and automotive [44,45] fields. The SMAs are characterized by their crystalline structure that undergoes solid-to-solid phase transformations. This crystalline structure is affected by two physical factors, temperature, and stress, causing SMA deformation. The transformation phase is called the martensite phase, which presents tetragonal, orthorhombic, or monoclinic crystalline structures with several possible variants, and the austenite phase is known for its cubic crystalline structure. Upon cooling and exerting the stress, the SMA deforms (martensite phase). Upon heating, it deforms again to its initial remembered shape (austenite phase). Moreover, another specialty of SMAs is their ability to undergo a reversible transitional solid–solid phase between the two phases [46,47,48]. SMAs have two specific and unique characteristics: the superelastic effect (SEE) and the shape memory effect (SME). Moreover, SMAs exhibit isotropic and anisotropic behaviors depending on the relation between the martensite volume fraction and equivalent transformation strain [49].

**Figure 1:**
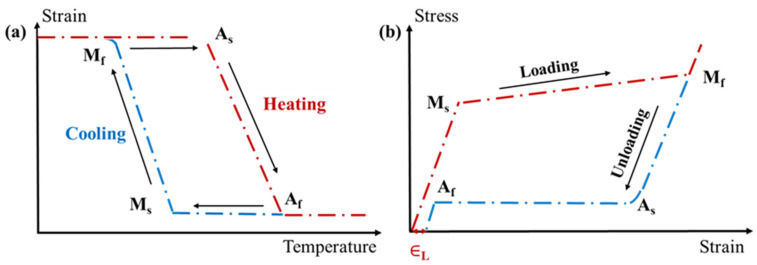
(**a**) The one-way shape memory effect of SMA wire. (**b**) Superelastic effect at a constant temperature. M_s_ and M_f_ are the start and finish martensitic phase temperatures, respectively; A_s_ and A_f_ are the start and finish austenitic phase temperatures, respectively.

The shape memory effect [50] demonstrates two pathways. The first is the ability of an SMA to recover the deformation by phase transitions induced by a thermal cycle. At a fully twinned-martensitic state (at a temperature below the final martensitic phase temperature M_f_), if the SMA is loaded by stress greater than or equal to the detwinned starting stress, then it remains strained (deformed), even upon unloading, since there is then a detwinned-martensitic state. This is known as the “detwinned” process. The SMA can then recover this deformation by heating to reach a whole austenite state (at a temperature above A_f_, see Figure 1a. Additionally, the SMA remains at its initial shape upon cooling below M_f_, but it moves back into a twinned martensite state. In a two-way SME, the SMA memorizes two shapes, namely, austenite and martensite, under certain conditions [51]. However, this property can be achieved by properly training the SMA, which means exerting many loaded cycles of two fixed states on the SMA to reach null plastic strain growth.

Superelasticity (pseudoelasticity) [52] is the ability of an SMA to undergo maximum stretching without residual strains (no remaining strain when no more stress is exerted) [12]. At temperatures above A_f_, if the critical M_f_ value of stress is exerted on an SMA, then a transition from austenite to a detwinned martensitic state occurs and causes deformation. Once it is unloaded, since the martensitic is not stable at a temperature greater than A_f_ without stress, it recovers its deformation, leading to a maximum residual strain ∈_L_ and transforming again to an austenite state (see Figure 1b). Superelastic behavior occurs during the phase transformation from martensite to austenite and austenite to martensite, which is induced by exerting threshold stress as an isothermal process.

Focusing on the SMA parameters, we define eight parameters, which are the four transformation temperatures (M_s_, M_f_, A_s_, A_f_), Young’s moduli during the two phases, and two stress-influenced coefficients, C_M_ and C_A_ [53]. In recent years, many studies on the numeric modeling of SMAs aim to predict behavior depending on the material’s characteristics. Commercial software such as Ansys [54] uses the Auricchio SMAs model, which accounts for superelastic and shapes memory behaviors. However, the process of building and computing the model takes a long time.

However, in [55], reinforcement learning (RL) on the MATLAB platform was used to study the behavior of a Cu-Ni-Ti SMA wire in real-time simulations by characterizing the major hysteresis loop (when the stress is enough to actuate the system) and minor hysteresis loop (when the SMA is not sufficiently actuated) of the shape memory effect strain–temperature relation. The experimental setup consisted of an SMA wire, an elastic spring, a voltage supply, a linear voltage differential transducer (LVDT) (strain sensor), a thermocouple (temperature sensor), a DAQ board, and alligator clips. The RL script on MATLAB interfaced through LabVIEW software to communicate within the experimental setup. The LabVIEW program converts the input voltage from the sensors to strain and temperature data that are sent to the MATLAB platform. RL determines the voltage needed to be applied on the SMA wire to reach the strain target. Therefore, at the same time, the strain–temperature relation is learned. The article concluded that RL could be an approach to control SMA wires based on more studies and experiments.

In addition, Rustighi et al. used an adaptively tuned vibration absorber (ATVA) for controlling an SMA [56]. TVA is a vibration control device, and ATVA is optimal for vibration tuning. The relation between ATVA and SMA can be represented by using the SMA stress and strain and the ATVA frequency and stiffness upon temperature variation. Different algorithms, such as proportional, proportional-plus-derivative PD, and fuzzy algorithms, can be used.

The research field of SMA studies proved their potential in various fields. However, there is a lack of advanced and non-traditional methods for modeling the SMA with low computational time and high accuracy, especially in real-time.

## 3. Description of Artificial Neural Networks

**Figure 2:**
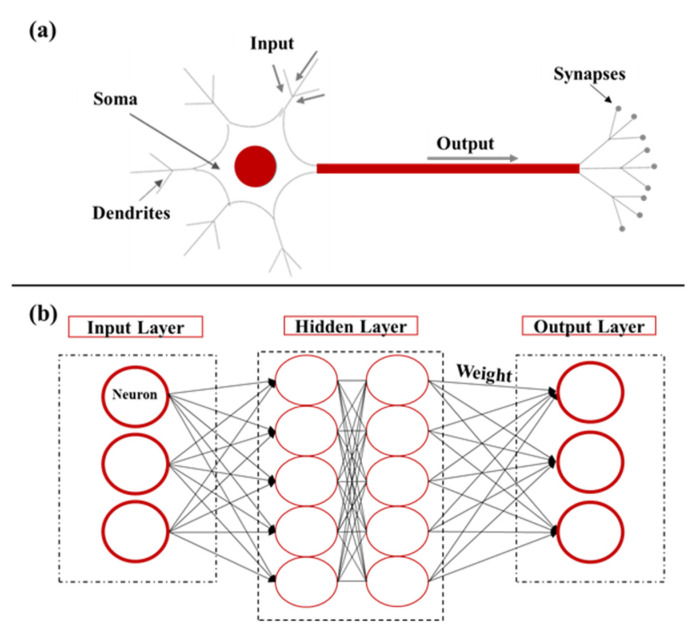
(**a**) Biological neuron architecture. (**b**) Artificial neural network architecture. (**a**) The labeled main parts of the neuron form the passage of the message from the input dendrites to the output synapses. (**b**) We labeled the main parts of an artificial neuron network for the usual three layers (input layer, hidden layer, and output layer).

Biological neuron functions have inspired the new programming and technological processes of ANNs [57,58,59,60]. For some biological knowledge, neurons, as cells of the nervous system and major information processing units, simulate the activities of the nervous system in the human brain. Neurons consist of the following: dendrites that receive input messages from other neurons, a soma (body cell) where the messages are transformed to action or result, an axon that conducts the output message from the soma away from the neuron, and synapses that permit the passage of messages from one neuron to another (connections) [61] (see Figure 2a).

There are different types of ANNs, such as clustering [62,63], time series, pattern recognition and forecasting [64], and fitting [65] NNs. ANNs can be differentiated by the number of neurons, the edges (connections of the neurons), the weights of connections, the number of hidden layers, and the functions of transformation and activation that take place in somas, as presented in Figure 2b [66]. A weight proves the significance of an information flow (arrows in Figure 2b), which means its effect.

**Figure 3:**
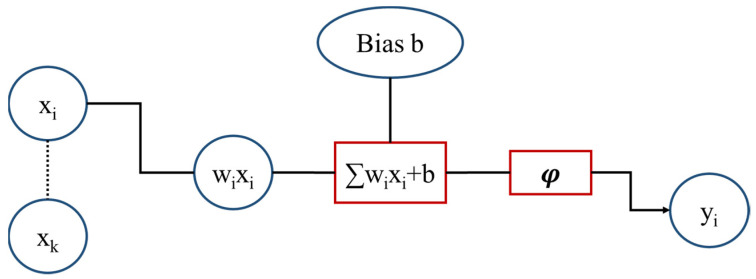
Network Functioning. X_i_ is the input parameter; wi is the weight; Σ is the transformation function, which can be the identity, b is the bias; and φ is the activation function.

As a description of the functioning of a typical numerical artificial neuron, the input Xi is multiplied by its weight w_i_. The transformation function Σ, which can be the identity, transforms the weighted inputs through the hidden layers by summing them with bias b. The results are activated using an activation function φ (commonly with a threshold). Finally, the output vector is formed, which can exit the work or input another ANN (see Figure 3). However, there are many different forms of activation functions; some of them are presented in Table 1. A shallow NN is a special NN consisting of one or two hidden layers, regardless of the type of functioning [67].

ANNs have been designed for many fields [68,69]. Particularly, the use of ANNs is attractive for real-time simulations. For instance, this technology can be used in robotic manipulator applications for real-time “comfortable” path planning in nonstationary environments [70]. Moreover, ANNs have been successful in gesture control for musical applications in pure data (PD) environments [71]. Controlling missile interception is an application based on ANNs that has been employed to perform real-time guidance [72]. In [73], the authors used an ANN to detect forest fires using a wireless network sensor in real-time.

Accordingly, ANN proved its high ability to model complex material systems with precise outputs and low computational time. ANNs and their training have successfully achieved up-to-date objectives that could enable them to perform real-time simulations.

## 4. SMA Forms and ANN Applications

SMAs are a class of smart materials. However, these materials are not simple to use in the engineering field because of the lack of a model to represent their nonlinear behavior and control their deformation in real-time.

Focusing on general real-time simulations, ANNs have been demonstrated in different studies and applications. Therefore, various studies concerning SMA simulation are based on ANNs. However, the behavior of an SMA depends on its forms, such as a single wire, ring, or reinforced concrete beams. Additionally, SMAs can be embedded in structures, such as manipulators with variable stiffness mechanisms [74], antagonistic actuators [75,76,77,78], or structures with multi-antagonistic mechanisms [79]. Moreover, these materials can be customized considering the type of application, including medical [5], robotic [38], civil engineering [40], aerospace [42], automotive [44], and other engineering [80] applications. For each studied problem, there could be a specific ANN.

SMAs based on deep learning can be classified according to their form and way of functioning. The classification includes linear systems of one wire (Section 4.1), systems with one wire and one spring (Section 4.2), magnetic SMAs (Section 4.3), SMA wires for rotatory actuation (Section 4.4), reinforced concrete (RC) beams (Section 4.5), porous SMAs (Section 4.6), rings and bars in self-centering and damping devices (Section 4.7).

### 4.1. Systems with a Wire for Linear Actuation

An SMA as a single wire was tested using ANN in a proportional-derivative (PD) position control system with a position sensor, as described in [80]. The wire was used as an actuator. The highlighted objective of the study was to control the position of the actuator by using the temperature, location, and electrical resistance of the actuator with optimal values. In [73], the authors controlled a Nitinol wire (diameter = 0.15 mm and length = 75 mm) using an inverse hysteresis model of the martensitic–austenitic transition. As mentioned before, the transformation between these two phases is nonlinear. Therefore, the NN is based on the LM algorithm designed to approach the second-order training speed (which uses a Hessian matrix depending on size and direction) and implemented in MATLAB [81]. For training the NN, 3000 epochs (the training parameter that refers to the number of passes of the entire training dataset) were found to be necessary to find a global minimum error of 2–3 µm.

In [82], an NN estimator was developed to control the position of an SMA actuator wire based on its generated load. The author’s main point is that this controlling method does not affect the actuator operator’s frequency.

However, one cited problem is the use of the position controller LVDT because it does not accurately represent the nonlinear variation in the SMA configuration. In [8], this point was considered by using electrical resistance (ER) feedback. The authors used a Ni-Ti single wire that was 228.6 mm in length and 0.381 mm in diameter. Its austenite-finish temperature was 90 °C. These researchers studied the dependence of strain responses and hysteresis on the ER to deduce the relationship between the ER and SMA pose. In this test, multilayer NNs consisting of two inputs (ER and a “tag” signal) and a 3-layer structure with one hidden layer of eight neurons were employed to obtain the proper displacement as an output. Hence, the approximate error was 7% because of the hysteresis feature, which can be explained by the heating time of the wire is higher than that needed for cooling it.

Moreover, ANNs can be used to analyze the properties of Ni-Ti SMA wire specimens produced by electrical discharge machining [83], including roughness, maximum peak to valley height, square roughness, and micro-hardness. A GRNN (general regression) with a multivariate hybrid approach VIKOR-Fuzzy logic system was used to optimize the machine’s setting parameters. The GRNN architecture had 54 datasets (60% for training, 20% for network validation, and 20% for testing) and predicted the machine’s responses with a ±5% error.

Recently, in [84], we modeled SMA systems using an NN, starting with a single SMA wire and ending with a common antagonistic SMA system (two SMA wires). The latter system consisted of two identical wires attached at a midpoint. Therefore, we learned the butterfly behavior of this SMA actuator using a long short term memory NN with a regression layer. As a result, the RMSE values and the computational time of the training do not exceed 2% and 3 min on average, respectively. The prediction times after the training are of the order of several ms.

### 4.2. Systems with One Wire and One Spring for Linear Actuation

An SMA and a bias spring act here as an actuator system using an NN, such as in [85]. These investigators evaluated the measurement of the shape recovery force of the fabricated SMA. The results of the NN prediction override that of the conventional equation.

In [86], the time response in the hysteresis behavior of a thin SMA wire with a diameter of 0.001 inches at different frequencies was predicted to cover the main and minor loops of the hysteresis. The experimental setup consists of a spring bias with a thin wire and a laser sensor with an applied current to heat the wire to achieve the phase transition and hysteresis effect. These researchers provided an innovative NN Jordan–Plus–Elman (Jordan–Elman network) nonlinear autoregressive exogenous (NARX) recurrent neural network (RNN). The NARX NN is a recurrent dynamic network with feedback connections enclosing several network layers. The NARX model is based on the linear ARX model, commonly used in time-series modeling. The next value of the dependent output signal y(t) is regressed on previous values of the output signal and an independent (exogenous) input signal. Experimental data were used to compare the NN Jordan–Elman architecture and the current NN Jordan–Plus method. Hence, when training the NNs with experimental chirp data (data as a signal whose frequency decreases or increases with time) as input, the RMSE error of the corresponding output of the Jordan–Elman network is less than that of the trained Jordan NARX network.

In [87], Song et al. controlled an SMA wire with a spring bias considering the forward and inverse hysteresis effect. These researchers modeled the displacement variation as a function of the applied voltage on the SMA to replace the position sensor. This was performed by training two NNs, which demonstrated a neural network inverse model for the feedforward controller and a neural network open-loop model for the tracking controller. Moreover, in [88], an NN with a sliding-mode-based robust feedback controller was employed to control Ni-Ti in both open- and closed-loop ways.

In addition to the issue of controlling the SMA wire and the steel spring, the SMA must be identified. Therefore, two approaches, namely, a hysteresis operator and a functional link artificial neural network (FLANN), were utilized. The FLANN and NARX models play different roles in identifying the system dynamically. The first approach detects the hysteresis behavior, and the second one approximates the system’s dynamics. The combination of the approaches is called HFLANN. In [89], first, the FLANN was modified, and its parameters were trained using particle swarm optimization (PSO) to identify the hysteresis behavior. Second, the system was controlled by using the model predictive controller-based neural network HFLANN. As a result, the error of testing FLANN as a predictor controller is less than or equal to 0.02 mm compared to the sinusoidal and multistep predictors (experimental results).

### 4.3. Magnetic SMA System

As a recent new application [90], a magnetic shape memory alloy (MSMA)-based actuator was studied, and its nonlinear hysteresis behavior was modeled. The authors built a fuzzy algorithm and a Takagi–Sugeno fuzzy neural network (TSFNN) model, improving the bacterial foraging algorithm (BFA). In other words, the NN was optimized by the modified bacterial foraging algorithm (MBFA). Hence, they proved the effectiveness of TSFNN and MBFA thanks to the inner nonlinear mapping function and self-adjustment that meet the needs of the nonlinear hysteresis effects. Accordingly, the authors compared the responses of training and optimization to those of the gradient descent algorithm (GDA).

In [91], an SMA and a steel spring were controlled using a magnetic approach. These authors trained a proportional–integral–differential PID NN by varying its weights using the backpropagation algorithm to characterize the forward and inverse hysteresis loops. As a result, the PID NN is tested with a 0.0073 mm maximum prediction error for only the major hysteresis loop in the first study and 0.0101 mm for both the major and minor hysteresis loops in the second study.

### 4.4. SMA Wire Systems for Rotatory Actuation

In addition, SMA can act as a rotatory manipulator actuator with the challenge of mastering severe hysteresis phenomena. In [92], the single-degree-of-freedom rotatory manipulator was controlled by employing NNs with high performance. Two controllers were used. The first one is a variable structure control (VSC) switch controller (a switch that actuates magnetic contactors and remote-operated controllers) with a hidden layer around the switching surface of the manipulator, and the second one is a neural plant model. Moreover, in [10], backpropagation NN (BPNN) direct control was developed with online learning control. This means that actuator position data were used to update the weight coefficients of the neural network. At the same time, the system controller was applied without previous training nor the inclusion of a hysteresis model. These authors work on a 1-DOF manipulator system actuated by a Flexinol shape memory alloy wire. The study aimed to predict its angular position. The three fixed parameters are the diameter and the original length of the Flexinol wire, and the temperature. The NN was trained by varying the weight factors, focusing on real-time for low computational cost and neural network direct control with online learning. The experiments were developed by moving the manipulator to different positions within the allowed range so that the different rotating angles were the NN’s inputs and the future position rotating angles the outputs. The rotational frequency and the torque were the studied factors after training the BPNN controller of the manipulator. As a result, a low computational time of controlling (20 ms) and a performance with a 0.83° error in the angular position at a frequency of 0.01 Hz were achieved. Additionally, the controller’s performance at an increased torque improved by 143%.

### 4.5. A Reinforced SMA Concrete Beam

Reinforced concrete (RC) beams are versatile composite materials that can hold high loads. SMAs for RC beams have been tested in just a few studies since reinforced SMA concrete beams can be damaged in earthquake events and are costly. Few studies have studied the properties of these materials with NNs [93,94]. The moment of inertia is a studied parameter of SMA RC that is a function of the reduction factor of the strength; this parameter reflects the deflection of the SMA. The reduction factor β is a function of the reinforcement ratio (the ratio of the beam’s area provided in a given section to the effective area of the section) and the reinforcement modulus of elasticity. In a previous study, such problems were solved using a neural network [95]. The nonlinear variation in β was addressed by training ANNs. The studied NN was a feedforward NN trained by a BP algorithm.

### 4.6. Porous SMAs

Porous SMAs (Ni-Ti) can be produced by thermal explosion or using the self-propagating high-temperature synthesis method (SHS) [96]. These materials are promising alternatives for medical applications [97] and machining [98]. Therefore, many investigations have been carried out to predict the mechanical and electrical properties of porous SMAs resulting from their unique features.

In [99], the authors trained a backpropagation neural network BPNN with one hidden layer and seven neurons. The effect of the heating rate (v), green density (D) (the higher the green density achieved, the finer the grain size), and reactant particle size of titanium (d) was tested on the mechanical properties to predict compressive yield stress and Young’s modulus. The designed BPNN modeled these proper porous SMA parameters with a low error of 2%.

In [98], porous Nitinol machining was characterized by applying the wire electrical discharge method (WEDM). The objective was to control the settings (as the servo voltage, pulse on time, pulse off time, current, and wire speed) and the responses (as the material removal rate (MRR) and surface roughness (Ra)). The authors modeled two connection types for NN multilayers, i.e., normal feed and full feedforward, to optimize the WEDM. These investigators trained the NN using three different learning algorithms, batch backpropagation BBP, quick prop QP, and incremental backpropagation IBP, with the number of neurons in the hidden layer varying from 5 to 20. There are five inputs (setting parameters) and two outputs (the machine’s responses). The best learning algorithm depends on the lowest average RMSE. For 20 neurons with a multilayer normal feed, the RMSE was 0.006705 using the IBP algorithm. For feedforward, the RMSE was 0.002294 using the BBP algorithms. In order to optimize the results of NNs, genetic algorithms were used, and the full feedforward connection provided the best results with respect to the experimental ones.

### 4.7. SMA Bars and Rings as Self-Centering and Damping Device

SMAs can play a role in self-centering and damping devices, which are performed by rings and bars. In [100], the behavior of dual (double) SMA rings was studied at different thermal states (martensitic and austenitic) by experimental and analytical methods using the FEM Ansys platform. In [101], the interest of SMA bars in self-centering was proven with a low mean residual storey drift ratio (the residual displacement ratio of the maximum displacement of the floor reference with respect to the floor below) of approximately 0.2% under a strong earthquake. In [102], the behavior of a Ni-Ti wire as a damper device was studied for use in civil engineering applications. In [103], the extreme load attenuation behavior of an SMA was investigated to determine the proper dimension and numbers of SMA Ni-Ti wires. In [104], the authors studied a bolt-form SMA bar to design seismic-resistant connections. All these studies depend on numerical and conventional methods. However, in [105], the Neuro-Fuzzy model was employed to study the self-centering and damping characteristics of the SMA when it was added to the sliding-type isolation system in a bridge to improve the seismic response. As a result, these authors verified that the superelastic behavior of the SMA occurs below a temperature of 10 °C.

Recently, in [106], thin plate structures were examined. These researchers demonstrated the performance of an actuator consisting of two SMA thin plates laminated by a thin aluminum plate. They modeled a vibratory frequency predictor using a BPNN optimized with a genetic algorithm and therefore succeeded in establishing a new dynamic stiffness model.

### 4.8. SMA Self-Sensing Systems

We have to highlight the potential of the SMA in the self-sensing field. In addition to actuating, SMA can play a sensor’s role, such as resistance sensor [107], angular position sensor [108], thermal sensor [109], or linear position sensor [110] simultaneously. Self-sensing is a capability that facilitates the controlling of the SMA system. NNs begin to be investigated for this purpose. One paper proved the self-sensing capability of an SMA wire with a linear spring using an Extended Kalman Filter based on ANN [111]. Moreover, for the antagonistic SMA wires system [112], the researchers used Deep NN (DNN) to study self-sensing. The DNN is composed of two LSTM layers and a middle feedforward layer. For a rotary manipulator system [108], they studied the self-sensing ability of the angular position using a shallow NN. They trained the model using the LM algorithm.

## 5. Discussion

In this discussion section, we highlight the main parameters of SMA, the main applications, the main neural networks, and the link between SMAs and NNs. It is developed consequently to the previous sections presenting the literature. The NNs are identified according to application types. Table 2, Table 3 and Table 4 are provided to summarize all the main aspects.

NNs obtain accurate results in SMA modeling, notably because they can learn the nonlinearity of the SMAs’ behavior. The procedure for modeling SMAs by NNs can be described in a few main steps: determine the precise form of the SMA, specify the modeling/controlled property and the controlling parameters of the SMA, decide the main characteristics of the NN, detect the proper learning or training algorithm, and optimize the results. Many NNs can learn the characteristics of SMAs by using various approaches and algorithms to handle the different properties of SMAs. Table 2 shows a classification of these properties, including mechanical, electrical, chemical, thermal, and dimensional ones. However, it is highly significant to distinguish between the modeling (controlled) properties and the controlling parameters of the SMA. The nonlinear behavior of SMAs reflects highly dependency between their properties and the parameters of the studies. For controlling the linear position of actuators, one has to focus on the actuator’s temperature, location, resistance, applied voltage, generated load, and the dimension of SMA. For the angular position, we should take into our account also its dependency on the torque and the rotational frequency. Porous SMAs modeling needs to predict the compressive yield stress and Young’s modulus properties depending on the heating rate, green density, and reactant particle size. In addition, in SMA machining, for controlling the material removal rate and surface roughness, the maximum peak to valley height, and the hardness, one must take into account the controlled settings such as servo voltage, pulse on time, pulse off time, current, and wire speed. For the RC beam SMAs, the NN depends on the reduction factor of the strength reinforcement modulus of elasticity and on the reinforcement ratio to model the moment of inertia.

Hence, we can highly realize the link between the SMA’s controlling and controlled parameters, its form, and the application. This classification is presented in Table 3. The shape of the SMA is an important parameter when choosing the properties of the NN since its behavior varies with its form and with the method of activation. As classified in Table 3, NNs can address various SMA shapes, including wires, porous materials, RC, bars, and rings.

Table 4 shows the different types of architectures for NNs used for studying SMA behavior, depending on the learning algorithms. However, most studies did not mention the reason for choosing the training or learning algorithm. Although the backpropagation algorithm is the most commonly used method for training neural networks and especially for feedforward NN, the characteristics and flowchart differ from one problem to another. In addition, the most commonly used NNs are feedforward NNs, NARX NNs, GRNNs, and Jordan Elman NNs. Feedforward NNs that use weight parameters are simpler than recurrent NNs, such as NARX NNs, that have cycles or loop connections between the nodes. The definitions of the NNs used are discussed in Table 4. The main effective parameters discussed in the studies are the number of epochs, hidden layers, neurons, weights, and input data [80,95]. Moreover, in many studies, new neural networks have been invented that are based on available NNs with some modifications, such as the innovative Jordan Plus Elman NN [44].

After considering the shape of SMAs and the properties to be studied, researchers must apply the different learning algorithms. Comparing the results of different NNs concerning the RMSE error, the best NN characteristics and algorithm for the considered problem can be deduced. However, the research does not end at this point; the maximization of the performance of the chosen NN is the next step. Different methods can be used including the following AI algorithms: Marquardt [80], Jordan–Plus–Elman NARX [86], back-propagation [10,81,91,98,99], batch back-propagation (BPP) [98], Quick Prop QP, incremental back-propagation (IBP) [98], fuzzy algorithm, bacteria foraging algorithm (MBFA) [90], GRNN (general regression) [83], particle swarm optimization (PSO) [89], VIKOR fuzzy algorithm, fuzzy algorithm genetic algorithm (GA), and gradient descent algorithm (GDA) [90]. We have seen in the descriptions that using metaheuristic optimization can help enhance the results. As a perspective, the link between AI techniques and metaheuristic optimizers could be generalized as in [113].

**Table 4:** Definition of neural networks used for modelling SMAs.

NN	Definition	Domain
Full feedforward NN	It treats the information only in one direction “forward” from the input nodes, through the hidden nodes (if any) to the output nodes without cycles or loops in the network [114]	Clustering Regression
Long short term memory NN	It has feedback connectors. Its unit consists of a cell, an input gate, an output gate and a forget gate. The gate is a threshold help NN distinguishing between using the identity connections over the stacked layers.	Prediction Classifying
Multilayer ormal feed NN	It is a full feedforward NN, but with multicomputational layers (multihidden layer).	Clustering Regression
Nonlinear autoregressive exogenous NN (NARX)	It is a recurrent NN that has loop connections between the nodes.	Time Series
NN estimator	It is NN that is based on an estimator, which is a technique or method that calculates an accurate result that depends on actual observations.	Prediction Classification
General regression (GRNN)	It has a radial-basis function layer and a linear layer [115]	Regression Approximation Classification
Proportional- integral-differential NN (PIDNN)	It is a dynamic feedforward network, a combination of neural networks with the PID control concept.	Controlling
Takagi–Sugeno fuzzy neural network (TSFNN)	It is a fuzzy system model that needs fewer inputs without the capability of handling online data [116]	Clustering
Functional link artificial intelligent NN (FLANN)	It is a single-layer higher-order class of an ANN [117]	Pattern Recognition Classification

Finally, the researcher should keep in mind the main targeted factors for training the NN, namely, low computational cost, direct control of the system by the NN, and low RMSE error. We note that the researcher finds difficulties when implementing NNs for SMAs. It is not easy since the threshold load, stress, and temperature, affect their nonlinear behavior. Particularly, more nonlinear data mean deeper NNs with more hidden layers and thus more complex architectures. In addition, the complexity of data is an issue in studies concerning SMAs and NN training that depends on various factors such as the complexity of the data and the data size.

## 6. Conclusions

In general, employing conventional methods such as numeric, FEM, and machining methods to model or fabricate shape memory alloy components is no longer sufficient because of their nonlinear behavior and the need for rapid computation time. Fortunately, ANNs achieve high performance in characterizing the shape memory alloy smart materials. NNs have various specificities that enable challenging to model the SMA nonlinear behavior in real-time. These specificities are notably the possibility of recovering complex and long-term patterns. The NN type, training algorithm, and dataset choices depend strongly on the considered problem and the corresponding SMA shape. The selection should be based on empirical knowledge according to the critical literature.

This paper presented a classification of the SMAs considering their properties and their forms. SMAs can have various shapes, e.g., wires, porous materials, RC beams, rings, and bars. Each one has specific features that can fit specific applications such as sensors, actuators, rotating manipulators, self-centering, medical, and conventional machining. The different properties of SMAs can act as inputs and outputs of the studied networks, highlighting the main characteristics of the SMAs. The hysteresis and shape memory effects surely involve nonlinear behavior. The studied properties are classified into mechanical, electrical, magnetic, thermal, chemical, and dimensional domains. Most of these properties can be characterized by various NNs. Theoretically, the proper choice of an NN depends on the type of the considered problem: classification, estimation, prediction, or simulation in real-time. To make this, we have referred to the studied literature and extracted the NNs type according to their considered problem. This classification has been presented. Table 4 showed the domains in which NNs are used according to the discussed articles. Note that NNs are trained by choosing the best weight of the inputs and inner neurons and the number of hidden layers. Activation functions play an important role in the training procedure. The value of the RMSE then proves the performance of the chosen network. Additionally, optimized training algorithms can decrease errors and provide more precise outputs and responses.

Regarding the studied literature, we have 15% of the scientific community that works on the feedforward NN type and 15% on BP. Concerning the shape of the SMA, 60% of the scientific community works are based on SMA wire as an actuator (linear and rotatory actuators), focusing on its mechanical and electrical parameters.

In summary, neural networks, part of the artificial intelligence domain, give satisfactory results. The NNs’ results validate the modeling of the shape memory alloy as a smart material. However, more studies and experiments for the nonlinear and nonconventional intrinsic behavior of these NNs (adequacy between the NN architecture and the SMA application) are needed to fit the requirements of engineering, medical, and robotics fields. More studies and experiments could further address the currently studied properties and nonstudied arrangements in complex devices. As perspectives, future research studies could more precisely discover the behavior and properties of SMAs, including solid–solid phase transformations with multi-structural units or complex forms.

## Figures and Tables

**Table 1:** Activation functions of the ANNs.

Activation Function	Equation
Step function	φ(x)=0 for x≤0, otherwise 1
Linear function	φ(x)=x
Rectified linear (ReLU)	φ(x)=max(0,x)
Hyperbolic tangent	$\varphi \left(x\right)=\frac{e^{x}+e^{-x}}{e^{x}-e^{-x}}$
Radial basis function	$\varphi \left(x\right)={\;e}^{x^{2}}$

**Table 2:** Classification of SMA’s application concerning its properties.

Type of Property	Description
Mechanical property	Roughness [98] Maximum peak to valley height [83] Square roughness [83] Microhardness [83] Load measurements [103] Position [10] Moment of inertia [95] Reduction factor betta [95] Green density (*D*) [99] Compressive yield stress [99] Density [99] Elastic modulus [99] Speed [99] Time response in the hysteresis behavior [86] Shape recovery force [85] Seismic response [104] Frequency [106] Strain response [84]
Thermal property	Austenite-finish temperature [8] Temperature [96] Heating rate (V) [99]
Chemical Property	Reactant particle size [99]
Electrical property	Servo voltage [98] Pulse on time [98] Pulse off time [98] Current [98] Electrical resistance (ER) [8]
Dimensional Property	Length of the wire [99] Cross-sectional area [99]
Magnetic Property	[90]

**Table 3:** Classification of studies as a function of SMA form and NN characteristics.

SMA Form	Application Type	NN Type	Training Method
**Wire**	Position Actuator	NN multilayer	Levenberg–Marquardt (LM) algorithm [80]
		NN Estimator	Parameter epochs: 3000 [80]
	Magnetic Actuator	Takagi–Sugeno fuzzy	MBFA and GDA algorithms [90]
	Rotatory-Manipulator: actuator	NN direct control with online learning	BP algorithm [10]
	Rotatory Manipulator: self-sensor	Shallow NN	LM algorithm [108]
	Linear Actuator with a spring bias	Nonlinear-Autoregressive Exogenous NARX NN	Jordan–Elman and Jordan–Plus–Elman algorithm [86]
		Proportional–Integral–Differential GRPID NN	Backpropagation algorithm [91]
		Functional Link Artificial Intelligent Neural Network	Particle-swarm optimization [89]
	Self-sensing with a spring bias	Shallow ANN	Extended Kalman Filter [111]
	Antagonistic System: Actuator	LSTM	[84]
	Self-sensor	DNN	DNN has two LSTM layers [88]
	Conventional Machining	General Regression	
		Forwarded	
	Conventional Machining	Multilayer normal feed	VIKOR FUZZY [83]
**Porous**	Medical	Multilayer normal feed Full feedforward	Batch Backpropagation [99]
	Earthquake Civil Damping Self-centering	Feedforward Backpropagation FFBP	Incremental Backpropagation [98]
		Vibrational control	Quick Prop algorithm QP [98] Genetic algorithms GA [98]
**Reinforced Concrete Beam**	Aircrafts	BPNN	Backpropagation algorithm [95]
**Ring and Bars**	Civil Damping Self-centering	Neuro_Fuzzy Model	[105]
**Plates**	Aircrafts	BPNN	Genetic algorithm [106]

## References

[1] Aïssa B., Memon N.K., Ali A., Khraisheh M.K. (2015). Recent Progress in the Growth and Applications of Graphene as a Smart Material: A Review. Front. Mater..

[2] Cao W., Cudney H.H., Waser R. (1999). Smart materials and structures. Proc. Natl. Acad. Sci. USA.

[3] Chaudhari R., Vora J.J., Patel V., López de Lacalle L.N., Parikh D.M. (2020). Surface Analysis of Wire-Electrical-Discharge-Machining-Processed Shape-Memory Alloys. Materials.

[4] Yang Y., Chen Y., Li Y., Chen M.Z.Q., Wei Y. (2017). Bioinspired Robotic Fingers Based on Pneumatic Actuator and 3D Printing of Smart Material. Soft Robot..

[5] Prokoshkin S., Brailovski V., Dubinskiy S., Zhukova Y., Sheremetyev V., Konopatsky A., Inaekyan K. (2016). Manufacturing, Structure Control, and Functional Testing of Ti–Nb-Based SMA for Medical Application. Shape Mem. Superelast..

[6] Isalgue A., Torra V., Lovey F., Terriault P., Carreras G., Soul H., Dieng L. (2010). Experimental study of damping in civil engineering structures using smart materials (NiTi-SMA). Application to stayed cables for bridges. Int. Rev. Mech. Eng..

[7] Tzou H., Lee H.-J., Arnold S.M. (2004). Smart Materials, Precision Sensors/Actuators, Smart Structures, and Structronic Systems. Mech. Adv. Mater. Struct..

[8] Ma N., Song G., Lee H.-J. (2004). Position control of shape memory alloy actuators with internal electrical resistance feedback using neural networks. Smart Mater. Struct..

[9] Zhang J.-J., Yin Y.-H., Zhu J.-Y. (2013). Electrical Resistivity-Based Study of Self-Sensing Properties for Shape Memory Alloy-Actuated Artificial Muscle. Sensors.

[10] Gómez-Espinosa A., Castro Sundin R., Loidi Eguren I., Cuan-Urquizo E., Treviño-Quintanilla C.D. (2019). Neural Network Direct Control with Online Learning for Shape Memory Alloy Manipulators. Sensors.

[11] Ölander A. (1932). An Electrochemical Investigation of Solid Caduim-Gold Alloys. J. Am. Chem. Soc..

[12] Zhang X.P., Liu H.Y., Yuan B., Zhang Y.P. (2008). Superelasticity decay of porous NiTi shape memory alloys under cyclic strain-controlled fatigue conditions. Mater. Sci. Eng. A.

[13] Qader İ.N., Kök M., Dagdelen F., Aydoğdu Y. (2019). A review of smart materials: Researches and applications. El-Cezeri J. Sci. Eng..

[14] Alsoruji G., Muthuramalingam T., Moustafa E.B., Elsheikh A. (2022). Investigation and TGRA based optimization of laser beam drilling process during machining of Nickel Inconel 718 alloy. J. Mater. Res. Technol..

[15] Jani J.M., Leary M., Subic A., Gibson M.A. (2014). A review of shape memory alloy research, applications and opportunities. Mater. Des..

[16] Carrera G.V.S.M., Branco L.C., Aires-de-Sousa J., Afonso C.A.M. (2008). Exploration of quantitative structure–property relationships (QSPR) for the design of new guanidinium ionic liquids. Tetrahedron.

[17] Xu S.X., Koko T.S. (2004). Finite element analysis and design of actively controlled piezoelectric smart structures. Finite Elem. Anal. Des..

[18] Manjaiah M., Narendranath S., Basavarajappa S. (2014). Review on non-conventional machining of shape memory alloys. Trans. Nonferrous Met. Soc. China.

[19] Kaya E., Kaya İ. (2019). A review on machining of NiTi shape memory alloys: The process and post process perspective. Int. J. Adv. Manuf. Technol..

[20] Chaudhari R., Vora J.J., Mani Prabu S.S., Palani I.A., Patel V.K., Parikh D.M., de Lacalle L.N.L. (2019). Multi-Response Optimization of WEDM Process Parameters for Machining of Superelastic Nitinol Shape-Memory Alloy Using a Heat-Transfer Search Algorithm. Mater. Basel Switz..

[21] Dick S. (2019). Artificial Intelligence. Harv. Data Sci. Rev..

[22] Liu Y., Zhao T., Ju W., Shi S. (2017). Materials discovery and design using machine learning. J. Mater..

[23] Mendizabal A., Márquez-Neila P., Cotin S. (2020). Simulation of hyperelastic materials in real-time using deep learning. Med. Image Anal..

[24] Bogue R. (2014). Smart materials: A review of capabilities and applications. Assem. Autom..

[25] Bogue R. (2012). Smart materials: A review of recent developments. Assem. Autom..

[26] Guo F., Guo Z. (2016). Inspired smart materials with external stimuli responsive wettability: A review. RSC Adv..

[27] Owusu-Danquah J.S., Bseiso A., Allena S. (2022). Artificial neural network models to predict the response of 55NiTi shape memory alloy under stress and thermal cycles. Neural Comput. Appl..

[28] Goldberg Y. (2017). Neural Network Methods for Natural Language Processing. Synth. Lect. Hum. Lang. Technol..

[29] Das S., Pegu H., Sahu K.K., Nayak A.K., Ramakrishna S., Datta D., Swayamjyoti S. (2020). Machine learning in materials modeling—Fundamentals and the opportunities in 2D materials. Synthesis, Modeling, and Characterization of 2D Materials, and Their Heterostructures.

[30] Butler K.T., Davies D.W., Cartwright H., Isayev O., Walsh A. (2018). Machine learning for molecular and materials science. Nature.

[31] Phillips C.L., Voth G.A. (2013). Discovering crystals using shape matching and machine learning. Soft Matter.

[32] Majid A., Khan A., Choi T.-S. (2011). Predicting lattice constant of complex cubic perovskites using computational intelligence. Comput. Mater. Sci..

[33] Lorente D., Martínez-Martínez F., Rupérez M.J., Lago M.A., Martínez-Sober M., Escandell-Montero P., Martínez-Martínez J.M., Martínez-Sanchis S., Serrano-López A.J., Monserrat C. (2017). A framework for modelling the biomechanical behaviour of the human liver during breathing in real time using machine learning. Expert Syst. Appl..

[34] Zakerzadeh M.R., Salehi H. Comparative Analysis of Some one-Dimensional SMA Constitutive Models for a Ni-Ti Wire for Shape Control Applications with Experimental Data. In Proceeding of the 20th International Conference on Adaptive Structures and Technologies.

[35] Prajna M.R., Antony P.J., Jnanesh N.A. (2018). Machine learning approach for flexural characterization of smart material. J. Phys. Conf. Ser..

[36] Farber E., Zhu J.-N., Popovich A., Popovich V. (2020). A review of NiTi shape memory alloy as a smart material produced by additive manufacturing. Mater. Today Proc..

[37] Morgan N.B. (2004). Medical shape memory alloy applications—The market and its products. Mater. Sci. Eng. A.

[38] Sreekumar M., Nagarajan T., Singaperumal M., Zoppi M., Molfino R. (2007). Critical review of current trends in shape memory alloy actuators for intelligent robots. Ind. Robot Int. J..

[39] Rodrigue H., Wang W., Han M.-W., Kim T.J.Y., Ahn S.-H. (2017). An Overview of Shape Memory Alloy-Coupled Actuators and Robots. Soft Robot..

[40] Song G., Ma N., Li H.-N. (2006). Applications of shape memory alloys in civil structures. Eng. Struct..

[41] Cladera A., Weber B., Leinenbach C., Czaderski C., Shahverdi M., Motavalli M. (2014). Iron-based shape memory alloys for civil engineering structures: An overview. Constr. Build. Mater..

[42] Hartl D.J., Lagoudas D.C. (2007). Aerospace applications of shape memory alloys. J. Sagepub.

[43] Exarchos D.A., Dalla P.T., Tragazikis I.K., Dassios K.G., Zafeiropoulos N.E., Karabela M.M., De Crescenzo C., Karatza D., Musmarra D., Chianese S. (2018). Development and Characterization of High Performance Shape Memory Alloy Coatings for Structural Aerospace Applications. Materials.

[44] Jani J.M., Leary M., Subic A. (2014). Shape Memory Alloys in Automotive Applications. Appl. Mech. Mater..

[45] Bellini A., Colli M., Dragoni E. (2009). Mechatronic Design of a Shape Memory Alloy Actuator for Automotive Tumble Flaps: A Case Study. IEEE Trans. Ind. Electron..

[46] Fang C., Zheng Y., Chen J., Yam M.C.H., Wang W. (2019). Superelastic NiTi SMA cables: Thermal-mechanical behavior, hysteretic modelling and seismic application. Eng. Struct..

[47] Chemisky Y., Duval A., Patoor E., Ben Zineb T. (2011). Constitutive model for shape memory alloys including phase transformation, martensitic reorientation and twins accommodation. Mech. Mater..

[48] Lovey F.C., Torra V. (1999). Shape memory in Cu-based alloys: Phenomenological behavior at the mesoscale level and interaction of martensitic transformation with structural defects in Cu-Zn-Al. Prog. Mater. Sci..

[49] Taillard K., Chirani S.A., Calloch S., Lexcellent C. (2008). Equivalent transformation strain and its relation with martensite volume fraction for isotropic and anisotropic shape memory alloys. Mech. Mater..

[50] Shape Memory Effect—An Overview|ScienceDirect Topics. https://www.sciencedirect.com/topics/chemistry/shape-memory-effect.

[51] Lexcellent C., Leclercq S., Gabry B., Bourbon G. (2000). The two way shape memory effect of shape memory alloys: An experimental study and a phenomenological model. Int. J. Plast..

[52] Barnes C. (1999). Innovations: Shape Memory and Superelastic Alloys. https://www.copper.org/publications/newsletters/innovations/1999/07/shape.html.

[53] Wang X., Hu G. (2005). Stress transfer for a SMA fiber pulled out from an elastic matrix and related bridging effect. Compos. Part Appl. Sci. Manuf..

[54] Divringi K., Ozcan C. (2016). Advanced Shape Memory Alloy Material Models for ANSYS. Ozen Eng. Inc..

[55] Kirkpatrick K., Valasek J. (2009). Reinforcement Learning for Characterizing Hysteresis Behavior of Shape Memory Alloys. J. Aerosp. Comput. Inf. Commun..

[56] Rustighi E., Brennan M.J., Mace B.R. (2005). Real-time control of a shape memory alloy adaptive tuned vibration absorber. Smart Mater. Struct..

[57] Wang S.-C., Wang S.-C. (2003). Artificial Neural Network. Interdisciplinary Computing in Java Programming.

[58] Abraham A. (2005). Artificial Neural Networks. Handbook of Measuring System Design.

[59] Kohli S., Miglani S., Rapariya R. (2014). Basics of artificial neural network. Int. J. Comput. Sci. Mob. Comput..

[60] Abdi H. (1994). A neural network primer. J. Biol. Syst..

[61] Hansen L.K., Salamon P. (1990). Neural network ensembles. IEEE Trans. Pattern Anal. Mach. Intell..

[62] Du K.-L. (2010). Clustering: A neural network approach. Neural Netw..

[63] Ghahari S., Queiroz C., Labi S., McNeil S. (2021). Cluster Forecasting of Corruption Using Nonlinear Autoregressive Models with Exogenous Variables (NARX)—An Artificial Neural Network Analysis. Sustainability.

[64] Dorffner G. (1996). Neural Networks for Time Series Processing. Neural Netw. World.

[65] Minasny B., McBratney A.B. (2002). The Neuro-m Method for Fitting Neural Network Parametric Pedotransfer Functions-Minasny. Soil Sci. Soc. Am. J..

[66] Haykin S. (2008). Neural Networks and Learning Machines.

[67] Albawi S., Mohammed T.A., Al-Zawi S. Understanding of a convolutional neural network. Proceedings of the 2017 International Conference on Engineering and Technology (ICET).

[68] Psaltis D., Sideris A., Yamamura A.A. (1988). A multilayered neural network controller. IEEE Control Syst. Mag..

[69] Xu L., Ren J.S., Liu C., Jia J. (2014). Deep Convolutional Neural Network for Image Deconvolution. Adv. Neural Inf. Process. Syst..

[70] Yang S.X., Meng M. (2000). Real-time Collision-free Path Planning of Robot Manipulators using Neural Network Approaches. Auton. Robot..

[71] Cont A., Henry C. (2004). Real-Time Gesture Mapping in pd Environment Using Neural Networks. NIME.

[72] Li Z., Xia Y., Su C., Deng J., Fu J., He W. (2015). Missile Guidance Law Based on Robust Model Predictive Control Using Neural-Network Optimization. IEEE Trans. Neural Netw. Learn. Syst..

[73] Yu L., Wang N., Meng X. Real-time forest fire detection with wireless sensor networks. Proceedings of the 2005 International Conference on Wireless Communications, Networking and Mobile Computing.

[74] Mekaouche A., Chapelle F., Balandraud X. (2018). A compliant mechanism with variable stiffness achieved by rotary actuators and shape-memory alloy. Meccanica.

[75] Yuan H., Chapelle F., Fauroux J.-C., Balandraud X. (2018). Concept for a 3D-printed soft rotary actuator driven by a shape-memory alloy. Smart Mater. Struct..

[76] Yuan H., Fauroux J., Chapelle F., Balandraud X. (2017). A review of rotary actuators based on shape memory alloys. J. Intell. Mater. Syst. Struct..

[77] Geaorges T., Brailovski V., Terriault P. (2012). Characterization and design of antagonistic shape memory alloy actuators-IOPscience. Smart Mater. Struct..

[78] Mohd Jani J., Leary M., Subic A. (2016). Designing shape memory alloy linear actuators: A review-Jaronie Mohd Jani, Martin Leary, Aleksandar Subic, 2017. J. Intell. Mater. Syst. Struct..

[79] Boufayed R., Chapelle F., Destrebecq J.F., Balandraud X. (2020). Finite element analysis of a prestressed mechanism with multi-antagonistic and hysteretic SMA actuation. Meccanica.

[80] Asua E., Etxebarria V., García-Arribas A. (2008). Neural network-based micropositioning control of smart shape memory alloy actuators. Eng. Appl. Artif. Intell..

[81] MATLAB Tutorial, Levenberg-Marquardt (Trainlm): Backpropagation (Deep Learning Toolbox). https://fr.mathworks.com/help/deeplearning/ref/trainlm.html.

[82] Senthilkumar P., Umapathy M. (2014). Use of load generated by a shape memory alloy for its position control with a neural network estimator. J. Vib. Control.

[83] Majumder H., Maity K. (2018). Application of GRNN and multivariate hybrid approach to predict and optimize WEDM responses for Ni-Ti shape memory alloy. Appl. Soft Comput..

[84] Hmede R., Chapelle F., Lapusta Y. (2022). Modeling the butterfly behavior of SMA actuators using neural networks. Comptes Rendus Mécanique.

[85] Lee H.J., Lee J.J. (2000). Evaluation of the characteristics of a shape memory alloy spring actuator. Smart Mater. Struct..

[86] Wang H., Song G. (2014). Innovative NARX recurrent neural network model for ultra-thin shape memory alloy wire. Neurocomputing.

[87] Song G., Chaudhry V., Batur C. (2003). A Neural Network Inverse Model for a Shape Memory Alloy Wire Actuator. J. Intell. Mater. Syst. Struct..

[88] Song G., Chaudhry V., Batur C. (2003). Precision tracking control of shape memory alloy actuators using neural networks and a sliding-mode based robust controller. Smart Mater. Struct..

[89] Tai N.T., Ahn K.K. (2012). A hysteresis functional link artificial neural network for identification and model predictive control of SMA actuator. J. Process Control.

[90] Zhang C., Yu Y., Wang Y., Zhou M. (2020). Takagi–Sugeno Fuzzy Neural Network Hysteresis Modeling for Magnetic Shape Memory Alloy Actuator Based on Modified Bacteria Foraging Algorithm. Int. J. Fuzzy Syst..

[91] Zhou M., Zhang Q. (2015). Hysteresis Model of Magnetically Controlled Shape Memory Alloy Based on a PID Neural Network. IEEE Trans. Magn..

[92] Nikdel N., Nikdel P., Badamchizadeh M.A., Hassanzadeh I. (2014). Using Neural Network Model Predictive Control for Controlling Shape Memory Alloy-Based Manipulator. IEEE Trans. Ind. Electron..

[93] Cao Y., Fan Q., Mahmoudi Azar S., Alyousef R., Yousif S.T., Wakil K., Jermsittiparsert K., Si Ho L., Alabduljabbar H., Alaskar A. (2020). Computational parameter identification of strongest influence on the shear resistance of reinforced concrete beams by fiber reinforcement polymer. Structures.

[94] Hannen J.C., Crews J.H., Buckner G.D. (2012). Indirect intelligent sliding mode control of a shape memory alloy actuated flexible beam using hysteretic recurrent neural networks. Smart Mater. Struct..

[95] Elbahy Y.I.E., Nehdi M.N., Youssef M.A.Y. (2010). Artificial neural network model for deflection analysis of superelastic shape memory alloy reinforced concrete beams. Can. J. Civ. Eng..

[96] Wisutmethangoon S., Denmud N., Sikong L. (2009). Characteristics and compressive properties of porous NiTi alloy synthesized by SHS technique. Mater. Sci. Eng. A.

[97] Ratner B.D., Hoffman A.S., Schoen F.J., Lemons J.E. (2004). Biomaterials Science: An Introduction to Materials in Medicine. San Diego Calif..

[98] Velmurugan C., Senthilkumar V., Dinesh S., Arul kirubakaran D. (2018). Artificial neural network prediction of wire electrical discharge machining properties on sintered porous NiTi shape memory alloy. Mater. Today Proc..

[99] Li Q., Yu J.-Y., Mu B.-C., Sun X.-D. (2006). BP neural network prediction of the mechanical properties of porous NiTi shape memory alloy prepared by thermal explosion reaction. Mater. Sci. Eng. A.

[100] Choi E., Nguyen H.D., Jeon J.-S., Kang J.-W. (2019). Self-centering and damping devices using SMA dual rings. Smart Mater. Struct..

[101] Qiu C., Fang C., Liang D., Du X., Yam M.C. (2020). Behavior and application of self-centering dampers equipped with buckling-restrained SMA bars. Smart Mater. Struct..

[102] Torra V. (2009). Damping in Civil Engineering Using SMA. The Fatigue Behavior and Stability of CuAlBe and NiTi Alloys. J. Mater. Eng. Perform..

[103] Torra V., Isalgue A., Auguet C., Carreras G., Lovey F.C., Terriault P., Dieng L. (2011). SMA in Mitigation of Extreme Loads in Civil Engineering: Damping Actions in Stayed Cables. Appl. Mech. Mater..

[104] Wang W., Fang C., Liu J. (2016). Large size superelastic SMA bars: Heat treatment strategy, mechanical property and seismic application. Smart Mater. Struct..

[105] Ozbulut O.E., Hurlebaus S. (2010). Evaluation of the performance of a sliding-type base isolation system with a NiTi shape memory alloy device considering temperature effects. Eng. Struct..

[106] Lu Y., Jiang J., Zhang J., Zhang R., Zhang Q., Zhou Y., Wang L., Yue H. (2022). A dynamic stiffness improvement method for thin plate structures with laminated/embedded shape memory alloy actuators. Thin-Walled Struct..

[107] Furst S.J., Crews J.H., Seelecke S. (2013). Stress, strain, and resistance behavior of two opposing shape memory alloy actuator wires for resistance-based self-sensing applications. J. Intell. Mater. Syst. Struct..

[108] Narayanan P., Elahinia M. (2016). Control of a shape memory alloy–actuated rotary manipulator using an artificial neural network–based self-sensing technique. J. Intell. Mater. Syst. Struct..

[109] Formentini M., Lenci S. (2018). An innovative building envelope (kinetic façade) with Shape Memory Alloys used as actuators and sensors. Autom. Constr..

[110] Tung A.T., Park B.-H., Liang D.H., Niemeyer G. (2008). Laser-machined shape memory alloy sensors for position feedback in active catheters. Sens. Actuators Phys..

[111] Gurung H., Banerjee A. (2016). Self-sensing SMA Actuator Using Extended Kalman Filter and Artificial Neural Network. Procedia Eng..

[112] Bhargaw H.N., Singh S., Botre B.A., Akbar S.A., Hashmi S.A.R., Sinha P. (2022). Deep Neural Network-Based Physics-Inspired Model of Self-Sensing Displacement Estimation for Antagonistic Shape Memory Alloy Actuator. IEEE Sens. J..

[113] Elsheikh A.H., Abd Elaziz M., Vendan A. (2022). Modeling ultrasonic welding of polymers using an optimized artificial intelligence model using a gradient-based optimizer. Weld. World.

[114] Feedforward Neural Networks 1. What Is a Feedforward Neural Network?. https://www.fon.hum.uva.nl/praat/manual/Feedforward_neural_networks_1__What_is_a_feedforward_ne.html.

[115] Generalized Regression Neural Networks-MATLAB & Simulink. https://www.mathworks.com/help/deeplearning/ug/generalized-regression-neural-networks.html.

[116] Wang D., Quek C., Ng G.S. MS-TSKfnn: Novel Takagi-Sugeno-Kang fuzzy neural network using ART like clustering. Proceedings of the 2004 IEEE International Joint Conference on Neural Networks (IEEE Cat. No.04CH37541).

[117] Naik B., Nayak J., Behera H.S., Abraham A. (2015). A self adaptive harmony search based functional link higher order ANN for non-linear data classification. Neurocomputing.

